# Silent Metastatic Pheochromocytoma: Clinical, Diagnostic, and Management Challenges in a Resource-Limited Setting

**DOI:** 10.7759/cureus.92737

**Published:** 2025-09-19

**Authors:** Bourhan Alrayes, Nashaat A Al-Shami, Anas Hamedat, Ahmad Khalil, Eman Hijazi, Bishr Shibani

**Affiliations:** 1 General Surgery, Islamic Hospital, Amman, JOR; 2 Internal Medicine, Islamic Hospital, Amman, JOR; 3 Oncology, Islamic Hospital, Amman, JOR; 4 Pathology and Microbiology, Islamic Hospital, Amman, JOR; 5 Science and Technology, Nottingham Trent University, Oxford, GBR

**Keywords:** atypical adrenal mass presentation, biochemically silent tumor, catecholamine-negative ppgl, chromogranin-positive tumors, lymph node metastasis, metastatic pheochromocytoma, neuroendocrine tumor, non-functional pheochromocytoma

## Abstract

Pheochromocytomas are rare neuroendocrine tumors arising from adrenal chromaffin cells, typically characterized by excess catecholamine secretion, which causes symptoms such as hypertension and palpitations. However, some pheochromocytomas and paragangliomas are non-functional and biochemically silent, complicating their diagnosis. Metastatic pheochromocytoma is uncommon and challenging to manage, especially in resource-limited settings.

We present the case of a 50-year-old man with progressive back pain, weight loss, weakness, and intermittent sweating. Imaging revealed bilateral adrenal masses with extensive lymphadenopathy and probable hypopharyngeal metastasis. Surprisingly, plasma and urinary metanephrines were normal. A biopsy of a cervical lymph node confirmed metastatic pheochromocytoma, demonstrating chromogranin and GATA3 positivity and a Ki-67 index of 20%. Despite multidisciplinary input, the patient’s lack of health insurance delayed treatment and resulted in death within one month.

This case highlights the diagnostic challenges of metastatic pheochromocytoma presenting with atypical symptoms and normal catecholamine levels. It emphasizes the need for comprehensive evaluation beyond biochemical tests, including imaging and histopathology. Additionally, it illustrates how socioeconomic barriers can limit access to timely care, adversely affecting outcomes. Awareness and early multidisciplinary management are essential to improving prognosis in metastatic pheochromocytoma.

## Introduction

Pheochromocytomas (PHEOs) are tumors originating from adrenomedullary chromaffin cells and are commonly associated with the production of catecholamines such as epinephrine, norepinephrine, and dopamine. However, paragangliomas (PGLs) arise from extra-adrenal chromaffin cells located either in the sympathetic paravertebral ganglia of the thorax, abdomen, and pelvis or in the parasympathetic ganglia associated with the glossopharyngeal and vagal nerves in the neck and skull base [1]. About 80%-85% of chromaffin-cell tumors are PHEOs, and 15%-20% are classified as PGLs [2]. The prevalence of both PHEO and PGL (PPGL) among hypertensive patients in outpatient settings is estimated to be between 0.2% and 0.6% [3,4].

Most PPGLs are characterized by catecholamine hypersecretion that may lead to increased cardiovascular morbidity and mortality [5,6]. Elevated levels of plasma or urinary fractionated metanephrines are considered key diagnostic markers for PPGLs because they confirm catecholamine overproduction [7]. Parasympathetic PGLs rarely metastasize (<10%). However, PHEOs have a metastatic rate of around 15%-20%, and sympathetic PGLs exhibit higher rates, with metastasis occurring in up to 70% of cases [8,9].

Predicting the malignant potential of PHEO remains challenging. Although grading systems have been proposed, histopathological features alone cannot reliably differentiate benign from malignant PPGLs [10]. As a consequence, the diagnosis of malignancy is based on the presence of distant metastasis [11]. Metastasis sites include regional and distant lymph nodes, bones, liver, and lungs [12,13]. As metastatic PPGLs are rare and aggressive in nature, early recognition, accurate diagnosis, and timely intervention are essential for improving patient outcomes.

## Case presentation

A 50-year-old Jordanian man presented with a chief complaint of progressive back pain that persisted for a month before admission. This pain was dull, intensified at rest, and was occasionally so severe that it disrupted his sleep. On questioning, the pain was found to be only partially alleviated by over-the-counter pain medication, paracetamol.

Detailed history explored that he experienced generalized weakness, a noticeably decreased appetite, and a significant unintentional weight loss of 14 kg within a month, decreasing from 100 kg to 86 kg. Strikingly, he reported episodes of excessive sweating, which he had been experiencing intermittently over the last two months.

His background consisted of being a father to three sons and being married. He had a significant smoking history of 25 pack-years. Additionally, the patient was uninsured, which would later be a significant obstacle in his management.

On clinical examination, the patient was conscious, alert, and fully oriented. His vital signs were noted as follows: body temperature was 36.1°C, heart rate was 71 bpm, respiratory rate was 18 breaths/min, blood pressure was 135/85 mmHg, and oxygen saturation level was 95% in room air. Interestingly, a palpable mass was felt in the left submandibular region.

Further investigations were initiated. Laboratory data from another hospital on December 17, 2022, revealed a slightly increased white blood cell count at 14.4 (4-11 × 10^3^/µL), with an elevated neutrophil count. Hemoglobin was decreased at 9.9 g/dL with a packed cell volume (PCV) of 30%. A high platelet count of 680 x 10^3^/µL was also noted. Renal parameters were mainly within the normal range, with a slightly lowered creatinine level. Other significant findings include an elevated ferritin level at 1,424 ng/mL (Table 1). Laboratory tests were repeated, showing negative results for any infective etiology (Table 2).

**Table 1:** Pre-hospital laboratory test The pre-hospital laboratory tests show normal results, including metanephrine in plasma and 24-hour urine collection, except for high ferritin. *Value is insignificant to mention

Test	Value	Reference range
White blood cell count (WBC)	14.4	4-11 × 10^3^/µL
Neutrophils	82	40%-75%
Lymphocytes	11	20%-45%
Monocytes	4	2%-10%
Eosinophils	3	1%-6%
Basophils	*	0%-1%
Hemoglobin (HB)	9.9	M: 13-17 g/dL
Packed cell volume (PCV)	30	M: 40%-50%
Red blood cell count (RBC)	3.7	M: 4.5-5.5 × 10^6^/µL
Mean corpuscular volume (MCV)	81	80-96 fL
Mean corpuscular hemoglobin (MCH)	26	27-32 pg
Mean corpuscular hemoglobin concentration (MCHC)	33	32%-35%
Red cell distribution width (RDW)	17	11.6%-14%
Platelet counts	680	150-450 × 10^3^/µL
Urea	28	10-50 mg/dL
Creatinine	0.5	0.7-1.2 mg/dL
Sodium	136	135-153 mmol/L
Potassium	4.7	3.5-5.3 mmol/L
Random blood glucose	100	RR UP to 199 mg/dL
Ferritin	1,424	25-350 ng/mL
Metanephrines 24 h urine	0.23	Up to 1 mg/24 h
Cortisol am serum	18.2	6.2-19 ng/mL
Metanephrine in plasma	30.8	Up to 65 pg/mL

**Table 2:** Assessment results A hospitalized assessment laboratory test showing a negative result for possible infective etiology was included in the differential diagnosis.

Test	Value	Reference range
White blood cell count (WBC)	8.4	4-11 × 10^3^/µL
Neutrophils	69	40%-75%
Lymphocytes	15	20%-45%
Monocytes	11	2%-10%
Eosinophils	2.4	1%-6%
Basophils	7.7	0%-1%
Hemoglobin (HB)	7.7	M: 13-17 g/dL
Packed cell volume (PCV)	23	M: 40%-50%
Red blood cell count (RBC)	2.9	M: 4.5-5.5 × 10^6^/µL
Mean corpuscular volume (MCV)	77	80-96 fL
Mean corpuscular hemoglobin (MCH)	26	27-32 pg
Mean corpuscular hemoglobin concentration (MCHC)	33	32%-35%
Red cell distribution width (RDW)	17	11.6%-14%
Platelet counts	798	150-450 × 10^3^/µL
Urea	21	10-50 mg/dL
Creatinine	0.5	0.7-1.2 mg/dL
Sodium	127	135-153 mmol/L
Potassium	4.9	3.5-5.3 mmol/L
Random blood glucose	100	RR UP to 199 mg/dL
C-reactive protein (CRP)	128	Less than 5
Erythrocyte sedimentation rate (ESR)	124	Less than 50 y: up to 15 mm/h
Prothrombin time (PT)	14.2	13.2 sec
International normalized ratio (INR)	1	-
Activated partial thromboplastin time (APTT)	31.9	26-36 sec
Calcium	8.8	8.6-10.2 mg/dL
Brucella	Negative	-
Hepatitis B surface antigen (HBsAg)	Negative	-
Hepatitis C virus antibody (HCV Ab)	Negative	-
Human immunodeficiency virus antigen/antibody combination for types 1 and 2 (HIV Ag/Ab 1+2)	Negative	-

**Figure 1:**
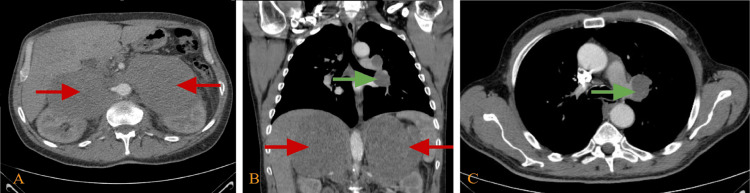
Chest and abdominal CT with intravenous contrast in the venous phase (A) An axial view of an abdominal CT scan showing two large hypodense regular masses in the adrenal glands bilaterally, causing an anterior displacement of abdominal organs with encasing of major vessels; these masses measure 14 x 11 x 17.7 cm and 12 x 10 x 15 cm in anterior-posterior, transverse, and craniocaudal dimensions (red arrows). (B) Coronal view of abdominal and thoracic CT scan showing two large hypodense regular masses in the adrenal glands bilaterally, causing a similar finding (red arrows), and a left hilar lymph node enlargement measuring 3.4 x 2.5 cm (green arrow). (C) Axial view of a thoracic CT scan showing a left hilar lymph node enlargement measuring 3.4 x 2.5 cm (green arrow).

Imaging studies, including an abdominal-pelvic CT with intravenous (IV) contrast, highlighted bilateral adrenal masses, with the right-sided mass being notably larger, measuring 11 x 8.6 cm (Figure 1). Further imaging revealed multiple enlarged lymph nodes in the thorax and the presence of two large hypoattenuating lobulated masses at the adrenal gland site.

**Figure 2:**
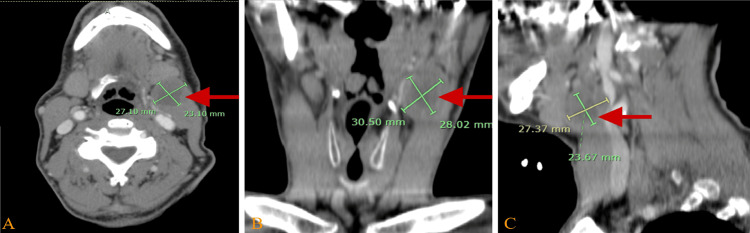
Neck CT with intravenous (IV) contrast (A) Axial view, (B) coronal view, and (C) sagittal view of a non-enhancing hypodense soft tissue lesion noted anterior to the left jugular vein, suggesting necrotic lymph nodes at the upper neck (group IIA level) with a 2.5 x 4 cm diameter compressing the surrounding structures such as the jugular vein and left submandibular gland.

Additionally, there was evidence of possible metastatic involvement in the left hypopharynx in the neck CT with IV contrast (Figure 2).

**Figure 3:**
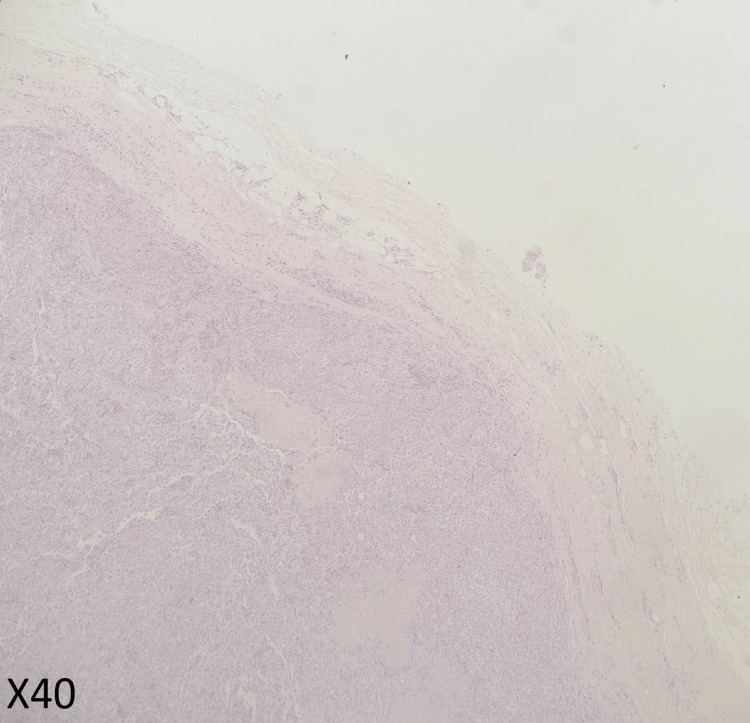
Histopathological studies with low power of H&E stain Lymph node is totally replaced by tumor cells growing in the sheet and nests, H&E x40.

**Figure 4:**
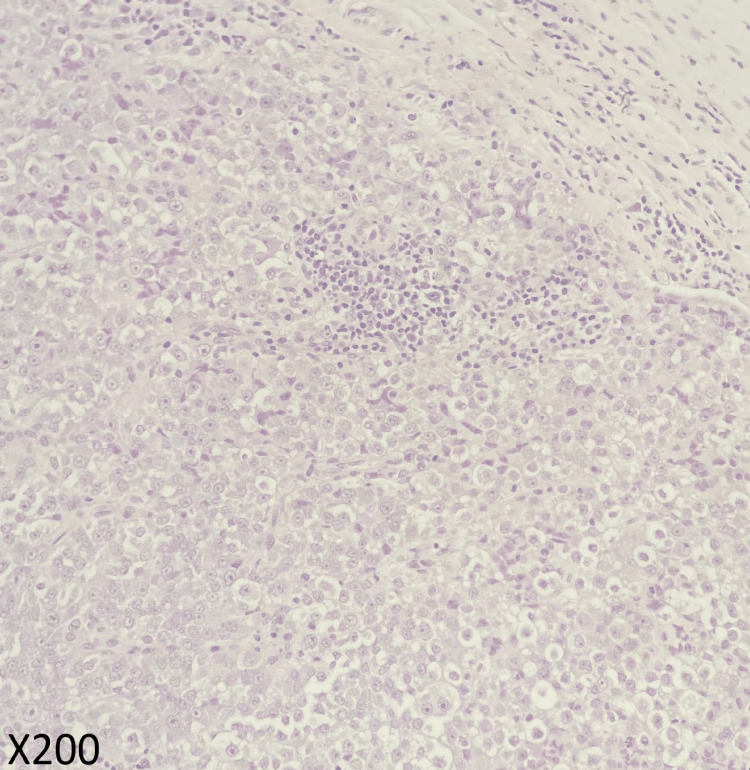
Histopathological studies with high power of H&E Tumor cells show ample cytoplasm with round nuclei and prominent nucleoli, H&E x200.

**Figure 5:**
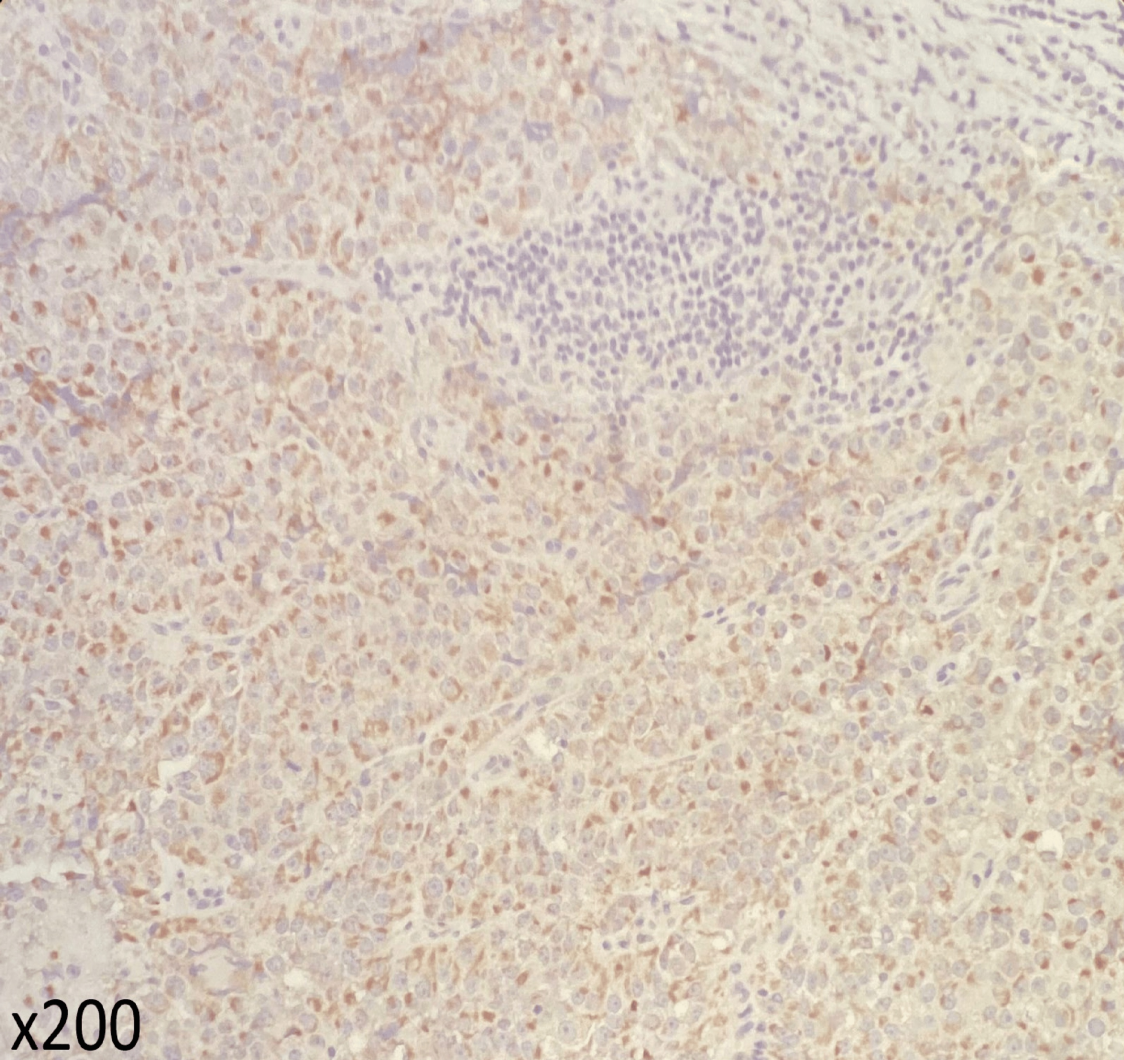
Histopathological studies with chromogranin stain Tumor cells showing positivity for chromogranin.

**Figure 6:**
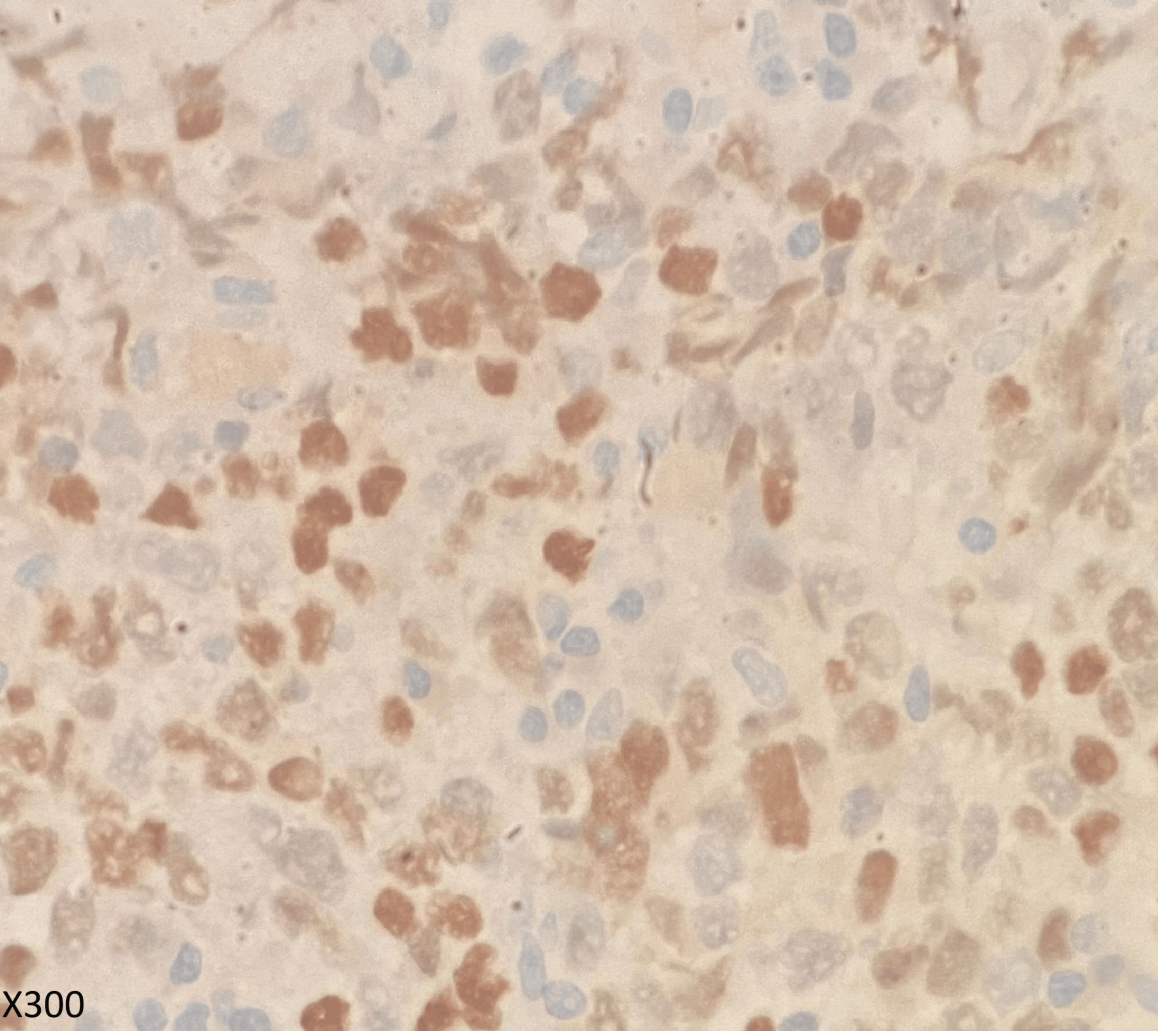
Histopathological studies with GATA stain Tumor cells showing positivity of GATA3.

Given these findings, a left cervical lymph node excisional biopsy was performed. The pathology returned showing fragmented lymph nodes with extensive areas of necrosis. The tumor cells were positive for chromogranin and GATA3 (Figures 3-6). A proliferation index of 20% was measured by Ki-67. Taking these findings with the radiological evidence, the patient was diagnosed with metastatic PHEO.

Regarding management, the patient was placed on IV fluids at 70 mL/hour and kept nil by mouth (NPO) pending further evaluation. He received red blood cell transfusions to manage his symptomatic anemia. As the patient was admitted under hematology, a gastroenterology consultation was done. Chemotherapy was recommended, but due to the patient's lack of insurance, the continuation of definitive treatment was not started. The constellation of clinical, radiological, and pathological findings pointed strongly toward a diagnosis of metastatic PHEO. However, the patient’s lack of insurance posed significant challenges in obtaining optimum care. The patient was admitted to a tertiary public hospital, where he passed away a month post-diagnosis without active treatment.

## Discussion

PHEO and PGL are rare conditions, affecting roughly one individual in every 300,000, with the average age at diagnosis being around 40 years [2,13]. Metastatic PHEOs and PGLs (MPPGs) are considered to be rare conditions, due to the fact that only up to 200 new cases are diagnosed each year in the United States [14]. The majority of PPGLs are associated with inherited genetic mutations, especially metastatic or multifocal cases. Up to 40% of PPGLs have a germline mutation, particularly in the SDHx gene cluster (SDHB, SDHD, SDHC), which correlates with a higher risk of malignancy, especially with SDHB mutations [15]. Genetic testing is currently recommended for all patients diagnosed with PPGLs, regardless of family history, to determine further management strategies and predict the prognosis [16].

PHEO and PGL have a wide range of clinical manifestations; these could present with symptoms of other medical conditions. The majority of these manifestations result from excessive catecholamine secretion and commonly include high blood pressure, headaches, heart palpitations, and feelings of anxiety [17]. From these, hypertension is the most frequently reported symptom and may occur either persistently or in sudden episodes. Some cases might also experience orthostatic hypotension [18]. Diagnosing PHEO/PGL typically includes clinical suspicion, biochemical confirmation, and imaging studies. Biochemical assessment focuses on the continuous release of catecholamines and their breakdown products, known as metanephrines [19,20]. Interestingly, this patient exhibited normal plasma and urinary metanephrines, which is atypical in large or metastatic PPGLs. In 10%-15% of PPGLs, this phenomenon may occur in non-functioning or low-secretory tumors [1]. In such cases, diagnosis often depends on radiological and histopathological findings rather than biochemical confirmation.

Anatomical and functional imaging modalities are helpful in the management of PHEO/PGL. Ga-68 DOTATATE, with other nuclear medicine techniques and radiotracers, plays a major role in diagnosis, management, and monitoring. Recently, efforts have been made to focus on determining the most sensitive radionuclide imaging agents for specific genetic subtypes [21,22].

GATA3 is a transcription factor commonly expressed in neuroendocrine tumors, including PHEOs and PGLs, and is useful in distinguishing these tumors from other malignancies [10,11]. Its expression supports neuroendocrine differentiation and may correlate with tumor aggressiveness and metastatic behavior [12]. While primarily a diagnostic marker, GATA3 may also play a role in tumor progression by influencing pathways involved in cell migration and epithelial-mesenchymal transition (EMT), contributing to metastatic potential [16].

Regarding management options for metastatic PPGLs, cytoreductive surgery is considered when feasible. Moreover, the main treatment is systemic chemotherapy with cyclophosphamide, vincristine, and dacarbazine (CVD regimen), 131I-MIBG therapy, and peptide receptor radionuclide therapy (PRRT) for somatostatin receptor-positive tumors [11,12]. Currently, targeted therapies, such as sunitinib, have also demonstrated a modest efficacy in SDHB-mutated tumors [23]. However, our patient's lack of health insurance access to these high-cost treatment modalities likely contributed to his rapid clinical decline.

This case underscores the importance of considering PPGL in patients presenting with atypical symptoms such as back pain, weight loss, and diaphoresis, especially when paired with constitutional symptoms. Furthermore, determinants of healthcare access, such as lack of insurance, are significant barriers to the timely diagnosis and treatment of rare endocrine malignancies. Awareness, early imaging, and a high index of suspicion can help in earlier intervention and, therefore, better outcomes.

## Conclusions

This case highlights the diagnostic and therapeutic challenges associated with metastatic PHEO, particularly when presenting with non-specific symptoms and normal catecholamine levels. The atypical biochemical profile underscores the importance of considering PPGLs in the differential diagnosis of patients with constitutional symptoms, unexplained weight loss, and radiologic evidence of adrenal masses-even in the absence of classical signs such as hypertension. Early recognition, multidisciplinary coordination, and improved access to healthcare resources are essential to optimize outcomes in such complex endocrine disorders.
